# Nitrate determines the bacterial habitat specialization and impacts microbial functions in a subsurface karst cave

**DOI:** 10.3389/fmicb.2023.1115449

**Published:** 2023-02-09

**Authors:** Xiaoyan Liu, Hongmei Wang, Weiqi Wang, Xiaoyu Cheng, Yiheng Wang, Qing Li, Lu Li, Liyuan Ma, Xiaolu Lu, Olli H. Tuovinen

**Affiliations:** ^1^State Key Laboratory of Geobiology and Environmental Geology, China University of Geosciences, Wuhan, China; ^2^School of Environmental Studies, China University of Geosciences, Wuhan, China; ^3^Department of Microbiology, Ohio State University, Columbus, OH, United States

**Keywords:** karst cave, subsurface biosphere, habitat specialization, nitrogen cycling, cooccurrence network, microbial function

## Abstract

Karst caves are usually considered as natural laboratories to study pristine microbiomes in subsurface biosphere. However, effects of the increasingly detected nitrate in underground karst ecosystem due to the acid rain impact on microbiota and their functions in subsurface karst caves have remained largely unknown. In this study, samples of weathered rocks and sediments were collected from the Chang Cave, Hubei province and subjected to high-throughput sequencing of 16S rRNA genes. The results showed that nitrate significantly impacted bacterial compositions, interactions, and functions in different habitats. Bacterial communities clustered according to their habitats with distinguished indicator groups identified for each individual habitat. Nitrate shaped the overall bacterial communities across two habitats with a contribution of 27.2%, whereas the pH and TOC, respectively, structured bacterial communities in weathered rocks and sediments. Alpha and beta diversities of bacterial communities increased with nitrate concentration in both habitats, with nitrate directly affecting alpha diversity in sediments, but indirectly on weathered rocks by lowering pH. Nitrate impacted more on bacterial communities in weathered rocks at the genus level than in sediments because more genera significantly correlated with nitrate concentration in weathered rocks. Diverse keystone taxa involved in nitrogen cycling were identified in the co-occurrence networks such as nitrate reducers, ammonium-oxidizers, and N_2_-fixers. Tax4Fun2 analysis further confirmed the dominance of genes involved in nitrogen cycling. Genes of methane metabolism and carbon fixation were also dominant. The dominance of dissimilatory and assimilatory nitrate reduction in nitrogen cycling substantiated nitrate impact on bacterial functions. Our results for the first time revealed the impact of nitrate on subsurface karst ecosystem in terms of bacterial compositions, interactions, and functions, providing an important reference for further deciphering the disturbance of human activities on the subsurface biosphere.

## 1. Introduction

Karst caves in central China are likely impacted by nitrogen leaching, mainly in the form of nitrate, originating from non-point sources such as agricultural industry and atmospheric deposition (Liao et al., 2018). Due to the thin soil layer in karst areas and the hydraulic connection between the surface water and groundwater, nitrate can enter the subsurface karst system through karst pipes. Increasing nitrogen inputs from anthropogenic sources can reduce biodiversity and enhance greenhouse gas fluxes (e.g., nitrous oxide, methane, carbon dioxide), leading to ecosystem degradation (Bobbink et al., 1998; Liu and Greaver, 2009; Van den Heuvel et al., 2011; Soons et al., 2017). However, the effects of nitrate infiltration on the microbial community composition and functions in karst caves remain poorly understood to date.

Nitrogen infiltration may weaken the carbon sink effect in karst caves through abiotic and biotic outcomes. On the one hand, the presence of exogenous nitrate enhances the dissolution of carbonate rock, releasing CO_2_ to the atmosphere (Spence and Telmer, 2005; Liu et al., 2008). On the other hand, methane-oxidizing bacteria (MOB), particularly the Upland Soil Cluster (USC), have been shown to oxidize atmospheric methane (1.8–2.0 ppm) in caves and help maintain caves as atmospheric methane sinks (Zhao et al., 2018; Cheng et al., 2021a). The net methane consumption may, however, be subject to competitive inhibition of the methane monooxygenase (MMO) by elevated concentration of NH_4_^+^ or through the stimulation of methanogenic archaea (Dunfield and Knowles, 1995; King and Schnell, 1998; Bodelier and Laanbroek, 2004).

Nitrogen deposition in soils has increased with the development of industry and agriculture (Shi et al., 2016; Zhou et al., 2017; Hu et al., 2021). Inputs of excessive N are known to cause soil acidification (Raza et al., 2020) and alter the composition and activities of soil microbial communities (Shi et al., 2016; Zhou et al., 2017; Hu et al., 2021) and thus their functions in natural ecosystems. Increased nitrate input can affect other soil chemical and biological properties.

It has been reported that the abundance of *Actinobacteria* and *Proteobacteria* typically increases with high N availability, whereas oligotrophic taxa such as *Acidobacteria* exhibit an opposite pattern (Fierer et al., 2012b; Hester et al., 2018; Nie et al., 2018). *Rhizobiales*, a group of soil borne diazotrophs belonging to the *Proteobacteria*, have been reported to be negatively correlated with elevated concentration of soil NH_4_^+^ and NO_3_^−^ (Che et al., 2018; Wang J. et al., 2021). Nitrogen fertilization decreased the bacterial diversity in soil perhaps by stimulating the growth of nitrophilous taxa and *via* competitive exclusion of other species (Campbell et al., 2010). Excessive nitrate input increases N_2_O emissions due to the decreasing pH, thus causing incomplete denitrification and making caves as greenhouse gas sources (Liu and Greaver, 2009; Van den Heuvel et al., 2011; Brenzinger et al., 2015). Nitrogen application has been shown to decrease the diversity of soil bacterial communities because of low pH levels (Yao et al., 2014), but opposite effects on bacterial diversity in natural ecosystems have also been reported in the literature. In general, microbiological changes upon nitrogen fertilization are difficult to deconstruct because of the complex interactions and associations in the soil–plant-rhizosphere-microbial community tangle.

In subsurface caves, environmental variables can significantly shape microbial communities. For example, microbial communities therein show a high habitat specialization and respond differently to environmental variables (Yang et al., 2021). TOC and pH have been reported to modulate bacterial communities in the Heshang Cave and the Xincuntun Cave across different habitats (Yun et al., 2016a; Cao et al., 2021). In the Luohandu Cave, for example, electrical conductivity and dissolved oxygen appear to influence microbial communities in dripping water and pool water, and those in sediments and weathered rocks are shaped by temperature, Ca/Mg, and sulfate (Yang et al., 2021), but these variables give little insight into understanding the underlying microbial biology. Our knowledge about how nitrate input impacts microbial compositions and functions in cave ecosystems is still far limited.

Cave microorganisms can be involved in nitrogen cycling *via* multiple ways. Dong et al. (2020) reported that in the Zhijin Cave bacteria participate in nitrogen fixation, denitrification, dissimilatory and assimilatory nitrate reduction, and comammox as indicated by PICRUSt (Dong et al., 2020). In addition, nitrification has also been reported in the Panlong Cave in the Guangxi Province (Zeng et al., 2022) and the Heshang Cave in the Hubei Province (Zhao et al., 2016). Several nitrate-reducing bacteria have been identified as keystone species in the co-occurrence network of cave ecosystems, such as *Gaiella* (Cheng et al., 2021a) and *Salinarimonas* (Dong et al., 2020). Autotrophic *Nitrospira* and *Nitrospirae* may play an essential role in sustaining the primary production through CO_2_ fixation coupled with nitrite oxidation in cave ecosystems (Ortiz et al., 2014; Lavoie et al., 2017; Kato et al., 2018; Ma et al., 2021). As a favorable electron acceptor, the input of nitrate can stimulate anaerobic substrate oxidations coupled with nitrate reduction and thus activate other geochemical processes (Xu et al., 2014).

Therefore, we hypothesize that nitrate input into the oligotrophic caves will significantly impact on subsurface microbial communities and stimulate the biogeochemical processes related to nitrogen as well as others. To test our hypothesis, we collected weathered rock and sediment samples in the Chang Cave, located in the western Hubei Province, which has a history of exposure to acid rain. Our results provide focus on variations in bacterial communities across different habitats and their responses to nitrate input, filling the gaps of anthropogenic impacts in the subsurface karst biosphere.

## 2. Materials and methods

### 2.1. Cave description

The Chang Cave (30°39′26.01″N, 109°58′27.49″E) is located in Jianshi County, Hubei Province, China, where has a typical subtropical monsoon climate (Supplementary Figure S1A). The average annual temperature is 13°C–18°C, and the average annual rainfall is 1,200–1,800 mm. The precipitation in late April to September accounts for about 70% of the total annual precipitation. The Chang Cave is a pristine cave with an average temperature of 15°C–18°C and close to saturated relative humidity. Multiple dripping points are observed here and there inside the cave, and the water is slightly alkaline with a pH of 7.5–8.5.

### 2.2. Sample collection

Samples of weathered rocks and loose sediments on the ground were collected with an interval of 50 m inward to the cave at 10 sampling sites in January 2020 (Supplementary Figure S1B). The weathered surface of the cave wall (0–1 cm depth) and loose sediments on the floor (to a depth of 2 cm) were collected. A five-point sampling strategy was exploited, and triplicate samples of weathered rocks and sediments were collected at each sampling site. All samples were transported under refrigeration within 24 h of collection to the Geomicrobiology Laboratory in China University of Geosciences (Wuhan). Samples were kept at −80°C upon arrival until analyzed.

### 2.3. Physicochemical analysis

All samples were freeze-dried (Alpha 12 LD freeze-dryer; Martin Christ, Osterode am Harz, Germany) and passed through a sterile 200-mesh sieve. Total organic carbon (TOC) content and C/N ratio were analyzed with a C-S analyzer (EA 4000, Analytik Jena AG, Jena, Germany) after acidification with 3 M HCl. One gram soil samples were mixed with 5 mL ultrapure water followed by 10 min vortex and subsequent centrifugation at 6,800 × *g* for 10 min (Yun et al., 2016b). Filtrates (0.22 μm membrane) were used for the pH measurement and dissolved ion analysis. The pH was measured with a multi-parameter water quality detector (HACH, Loveland, CO). The analysis of dissolved anions and cations was performed using anionic chromatography (ICS-600, Thermo Scientific, Waltham, MA) and ICP-OES (iCAP 7,600+, Thermo Scientific), respectively.

### 2.4. DNA extraction and 16S rRNA gene sequencing

Total nucleic acids were extracted from 0.5 g dry weight samples (freeze-dried and sieved through 200 mesh) using the MoBio PowerSoil DNA Kit (Qiagen, Redwood City, CA) following the manufacturer’s instructions. The concentration and quality of DNA were measured by a Nanodrop 2000 spectrophotometer (ND2000; Thermo Scientific) and visualized by 2% agarose gel electrophoresis. Bacterial diversity was examined *via* high throughput sequencing of the 16S rRNA genes with the primer set of 338F (5′-ACTCCTACGGGAGGCAGCA-3′) and 806R (5′-GGAC TACHVGGGTWTCTAAT-3′) on the Illumina Miseq platform (Shanghai Personal Biotechnology, Co., Ltd., Shanghai). The thermal cycling steps of the 16S rRNA genes were an initial denaturation at 98°C for 2 min, followed by 25 cycles of 98°C for 15 s, 52°C for 30 s, 72°C for 30 s and a final extension at 72°C for 5 min.

### 2.5. Sequence analysis

Raw sequence data were quality filtered and analyzed using QIIME 2 (Bolyen et al., 2019). Reads were processed by removing barcodes, primers, and low-quality sequences (with an average quality score < 30), in which chimera removed with DADA2 plugin. Subsequently ASVs (Amplicon Sequence Variants) representative sequences and feature tables were generated to annotate in the SILVA database release 134 (DeSantis et al., 2006). These samples were resampled to the same level of sequencing to avoid the impact of sequencing depth on microbial communities.

### 2.6. Statistical analysis

Alpha diversities were conducted in QIIME2 (Bolyen et al., 2019). Beta diversity analyses were performed to explore the differences in bacterial composition across habitats and sampling sites, which were visualized by non-metric multidimensional scaling (NMDS) based on the Bray–Curtis dissimilarity matrix using the vegan package (Oksanen et al., 2018) in R (R Core Team, 2020). Plots of linear regression were used to reveal the bacterial diversity along the cave depth using the R package ggplot2 (Wickham, 2016). The LEFSe analysis^1^ was used to construct the linear discriminant analysis (LDA) model to find out significant differences in bacterial taxa between weathered rocks and sediments (Segata et al., 2011). The analyzed geochemical parameters were assessed for the variable inflation factor (VIF) using the Vegan package of R Project to make all the remaining environmental variables exhibited maximum VIF values of no higher than 10 (Yu et al., 2016). Redundancy analysis (RDA) was performed to determine the most significant physicochemical parameters that shaped composition and structure of bacterial communities using CANOCO 5.0. Mechanistic study of the effect of nitrate on the α-diversity and β-diversity was conducted using piecewise structural equation simulation (piecewiseSEM) method with the R package piecewiseSEM (Lefcheck and Freckleton, 2015). The genera present in more than 20% of samples with a relative abundance of >0.05% were retained, and those significantly associated with nitrate (*r* > |0.5|, *p* < 0.05) were then used to construct the nitrate interaction networks. Co-occurrence network analysis of significant taxon may help to explore the structure of complex microbial communities across spatial or temporal gradients. Only ASVs with a proportion above 0.05% across all samples and occurring in more than 20% of samples were retained in co-occurrence networks. The co-occurrence network was constructed based on the Spearman rank correlations with a coefficient > |0.7| and a *p*-value <0.01 (Liu et al., 2020). Visualization of the robust pairwise correlations of the ASVs was implemented by Gephi (version 0.9.2) software (Bastian et al., 2009). According to within-module connectivity (Zi) and among-module connectivity (Pi) thresholds, all nodes were classified into four groups: peripherals (Zi ≤ 2.5 and Pi ≤ 0.62), connectors (Zi ≤ 2.5 and Pi > 0.62), module hubs (Zi > 2.5 and Pi ≤ 0.62), and network hubs (Zi > 2.5 and Pi > 0.62; Olesen et al., 2007), where connectors, module hubs, and network hubs were considered as keystone taxa in the network (Zhou et al., 2010; Fan et al., 2018). The metabolic function profiles were predicted using the FAPROTAX (Louca et al., 2016) and the R package Tax4Fun2 (Wemheuer et al., 2020). Histogram of the KEGG level-3 functional pathways and nitrogen metabolism genes and heatmaps of functions predicted by FAPROTAX were generated in R (R Core Team, 2020). Statistical analysis of metagenomic profiles (STAMP) was used to analyze the differential metabolic profiles between the weathered rocks and sediments (Parks et al., 2014).

## 3. Results

### 3.1. Physicochemical properties of samples in the Chang Cave

The pH values of weathered rocks and sediments were significantly different (*p* < 0.05), but both were slightly alkaline (pH 7.69–9.21 in weathered rock versus pH 7.78–8.42 in sediments; Table 1). TOC concentrations in sediments were higher than those in weathered rocks from S1 to S5, while the opposite pattern was observed in S6–S10. The C/N ratios in sediments were highly variable and the average value (5.85 ± 2.18) were higher than that in weathered rocks (5.25 ± 0.89). The nitrate concentrations in weathered rock samples (mean 1152.95 mg·kg^−1^) were significantly higher than those in sediments (mean 348.78 mg·kg^−1^) as proved by Kruskal-Wallis test (*p* < 0.01; Table 1).

**Table 1:** Physicochemical properties of samples collected from weathered rocks and sediments in the Chang Cave, Hubei province, central China.

	Na^+^ (mg·kg^−1^)		K^+^ (mg·kg^−1^)		Ca^2+^ (mg·kg^−1^)		NO_3_^−^** (mg·kg^−1^)		pH*		C/N ratio		TOC (%)	
	WR	S	WR	S	WR	S	WR	S	WR	S	WR	S	WR	S
S-1	1.69 ± 2.39	50.02 ± 2.84	36.79 ± 8.08	159.34 ± 7.06	294.66 ± 16.55	1374.80 ± 50.09	396.09 ± 67.93	277.34 ± 15.07	8.27 ± 0.07	7.86 ± 0.02	5.46	4.14	0.29	0.45
S-2	21.68 ± 16.25	/	530.95 ± 16.66	73.04 ± 3.15	1162.73 ± 66.17	113.18 ± 5.33	5156.01 ± 33.87	222.17 ± 18.77	7.69 ± 0.03	8.19 ± 0.03	4.81	8.00	0.83	0.92
S-3	0.79 ± 1.11	3.81 ± 3.86	22.56 ± 1.41	75.53 ± 80.26	96.66 ± 5.18	394.97 ± 412.67	27.60 ± 15.69	150.35 ± 15.85	8.11 ± 0.04	8.01 ± 0.22	5.06	8.70	0.70	1.02
S-4	2.47 ± 1.79	0.09 ± 0.13	70.96 ± 2.25	25.83 ± 1.30	281.82 ± 3.35	103.54 ± 0.83	852.45 ± 37.04	8.19 ± 10.95	7.93 ± 0.09	8.24 ± 0.02	4.52	9.57	1.25	1.24
S-5	32.20 ± 35.14	3.17 ± 0.44	61.38 ± 7.26	95.69 ± 0.81	379.31 ± 4.59	134.44 ± 0.15	1378.61 ± 81.61	247.92 ± 35.53	7.98 ± 0.02	8.26 ± 0.05	5.80	6.48	0.57	0.70
S-6	0.14 ± 0.20	11.58 ± 0.51	10.58 ± 1.36	19.22 ± 2.76	88.46 ± 0.34	48.88 ± 0.26	2.86 ± 2.05	62.90 ± 24.13	8.24 ± 0.06	8.47 ± 0.14	4.55	4.81	0.45	0.35
S-7	20.47 ± 0.37	18.12 ± 0.33	75.54 ± 0.14	38.28 ± 0.14	648.86 ± 2.86	112.50 ± 0.81	1256.47 ± 7.10	166.87 ± 9.21	7.87 ± 0.09	8.34 ± 0.04	4.36	5.21	0.36	0.32
S-8	3.31 ± 0.34	30.88 ± 0.66	13.57 ± 0.64	16.90 ± 1.89	86.63 ± 26.6	119.09 ± 1.27	100.95 ± 1.20	98.94 ± 11.30	9.21 ± 0.07	8.42 ± 0.02	7.58	2.29	0.07	0.11
S-9	6.83 ± 0.82	13.09 ± 0.24	26.87 ± 0.22	24.60 ± 0.66	197.34 ± 0.42	177.16 ± 0.42	287.53 ± 15.03	182.24 ± 25.43	7.90 ± 0.08	8.29 ± 0.06	5.34	5.12	0.43	0.37
S-10	62.25 ± 8.06	29.38 ± 10.62	24.17 ± 17.11	11.22 ± 8.91	1020.38 ± 103.35	2067.21 ± 92.72	2070.92 ± 23.55	2070.92 ± 1.02	7.84 ± 0.08	7.78 ± 0.05	5.04	4.15	0.60	0.23

The results with 0.01 < *p* < 0.05 and *p* < 0.01 were, respectively, marked with * and **. /: below the detection limit.

### 3.2. Habitat specialization of bacterial communities

**Figure 1:**
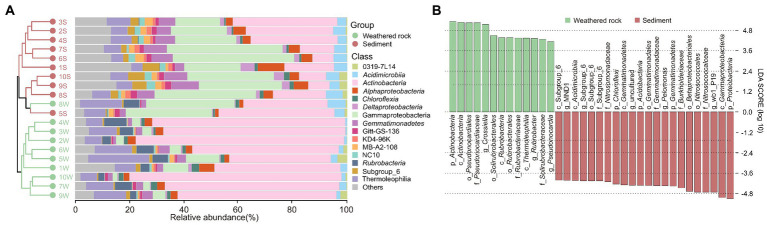
Compositions of bacterial communities at the class level **(A)** and microbial indicator groups in weathered rocks and sediments as indicated by least discriminant analysis with a LDA score > 4 **(B)** in the Chang Cave, Hubei province, China.

At the class level, *Actinobacteria* was dominant in weathered rocks (Figure 1A), with relative abundance ranging from 30.96% to 77.77%, followed by *Thermoleophilia* with a relative abundance of 6.13% to 22.44%. In sediments, *Gammaproteobacteria* and *Actinobacteria* dominated bacterial communities with minor amounts of NC10 groups (0.38%–3.08%). The abundance of *Rubrobacteria* (2.26%–8.35%) in weathered rocks was significantly higher than that in sediments (0.05%–0.33%).

Least discriminant analysis effect size distinguished indicator taxa in weathered rocks and sediments from the phylum to genus levels (Figure 1B). At the phylum level, *Actinobacteria* was specific to weathered rocks, whereas *Chloroflexi*, *Acidobacteria*, *Gemmatimonadetes*, and *Proteobacteria* were associated with sediments. *Pseudonocardiales*, *Solirubrobacterales*, and *Rubrobacterales* were indicator orders in weathered rocks, and subgroup_6, *Gemmatimonadates*, *Betaproteobacteriales*, and *Nitrosococcales* were indicators in sediments. *Crossiella*, *Rubrobacter,* and *Pseudonocardia* were indicator genera in weathered rocks, whereas MND1, subgroup_6, *Pelomonas*, and wb1_R19 were in sediments.

**Figure 2:**
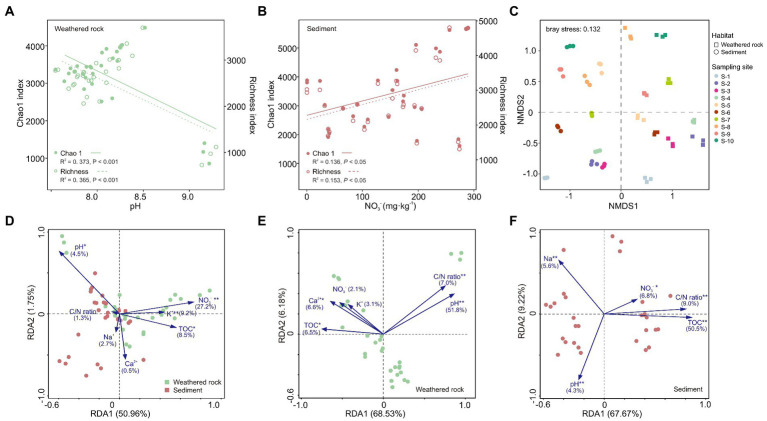
Bacterial diversities in weathered rock and sediment samples and redundancy analysis (RDA) of bacterial communities and environmental variables in the Chang Cave, Hubei province. **(A)** Linear regression analysis of alpha diversity indexes and pH in weathered rocks. Solid circles and solid lines: Chao1 index; hollow circles and dashed lines: Richness index. **(B)** Linear regression analysis of alpha diversity indexes and nitrate in sediments. **(C)** Non-metric multidimensional scaling (NMDS) ordination plot of bacterial communities based on Bray-Curtis dissimilarities. The squares represent weathered rock, the circles represent sediments, and the colors distinguish sampling locations. Redundancy analysis between environmental variables and bacterial communities at the class level in all samples **(D)**, weathered rocks **(E)** and sediments **(F)**. Significance level: *p* < 0.05, *; *p* < 0.01, **; *p* < 0.001, ***. Green: weathered rocks; red, sediments.

Overall, the alpha diversity in sediments was significantly higher than that in weathered rocks as indicated by alpha diversity indexes (Supplementary Figure S2). The alpha diversity (Chao1 and Richness) indexes of weathered rocks were significantly negatively correlated with pH (Figure 2A), and those in sediments positively correlated with nitrate (Figure 2B). Bacterial communities in weathered rocks and sediments were clearly separated by the NMDS ordination plot (Figure 2C). NMDS1 distinguished bacterial communities in different habitats. Bacterial communities in sediments were located in the second and third quadrants and those in weathered rocks in the first and fourth quadrants. Bacterial communities within an individual habitats were separated along NMDS2 by different sampling sites (Figure 2C).

### 3.3. Environmental impacts on bacterial community structures

The pH and concentrations of TOC, nitrate, and K^+^ were demonstrated to significantly impact bacterial communities in caves with a contribution of 27.2% by NO_3_^−^ (Figure 2D) across different habitats. As for each individual habitats, pH and TOC were, respectively, the most important environmental factors shaping the community structure in weathered rocks with an explanation of 51.8% (Figure 2E) and with an explanation of 50.5% in sediments (Figure 2F). In addition, NO_3_^−^, pH, C/N ratio and Na^+^ also significantly impacted bacterial communities in sediments.

**Figure 3:**
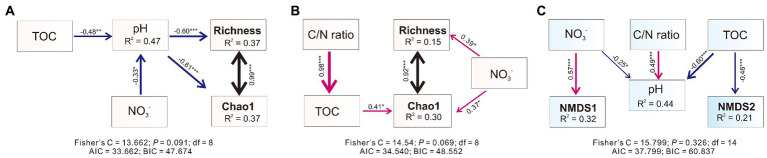
Piecewise structural equation modeling (piecewiseSEM) describing the effect of multiple physiochemical parameters on the microbial alpha [in weathered rocks **(A)**, sediments **(B)**] and beta **(C)** diversities. Numbers adjacent to arrows are indicative of the effect size of the relationship. The width of the arrows is proportional to the strength of the relationship. Red arrows indicate a significant positive correlation, while blue arrows indicate a negative relationship. *R*^2^ denote the proportion of variance explained by the predictors. Significance level: *p* < 0.05, *; *p* < 0.01, **; *p* < 0.001, ***.

Piecewise structural equation modeling (piecewise SEM) analysis revealed that the pH significantly and negatively shaped the alpha diversity (Chao1 and Richness indexes) of bacterial communities in weathered rocks and explained 37% of the variation (Figure 3A). Both NO_3_^−^ and TOC negatively impacted bacterial communities indirectly *via* influencing the pH (Figure 3A). Nitrate was significantly and positively correlated with Chao1 and Richness indexes in sediments (Figure 3B). In addition, TOC and Chao1 indexes also showed a significant positive correlation. The improved piecewise SEM model explained 30% and 15% variation in Chao1 and Richness indexes in sediments, respectively (Figure 3B). Structural equation model showed that nitrate positively correlated with NMDS1 and explained 32% of its variation, which distinguished bacterial communities in different habitats. TOC was negatively related to NMDS2, and explained 21% of the variation, which distinguished bacterial communities at different sampling sites within each habitat (Figure 3C).

**Figure 4:**
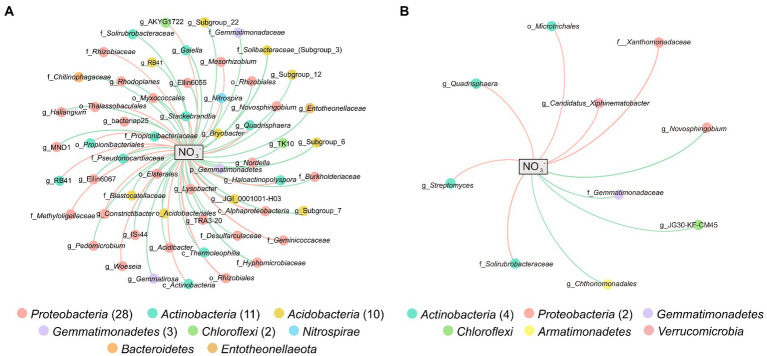
Networks between microbial genera and NO_3_^−^ in weathered rocks **(A)** and sediments **(B)**. Nodes are colored by microbial phyla, and the connection edge represents a significant correlation (|*r*| > 0.5, *p* < 0.05) with NO_3_^−^ based on pairwise Spearman’s correlations. Positive correlations are in red and negative correlations are in green.

Networks were constructed between bacteria and NO_3_^−^ to further explore the potential impact of NO_3_^−^ on specific microbial groups. In total, 57 and 10 genera with significant Spearman’s correlation (|*r*| > 0.5, *p* < 0.05) with NO_3_^−^ were included in the network of weathered rock (NW) and sediment (NS), respectively (Figures 4A,B). There were 20 (35.09%) and 6 (60%) genera showed positive correlations with NO_3_^−^ in NW and NS, respectively. Taxonomically, the 57 genera mainly belonged to *Proteobacteria* (28), *Actinobacteria* (11), *Acidobacteria* (10), *Gemmatimonadetes* (3), and *Chloroflexi* (2) in NW, whereas the 10 genera in NS were mainly affiliated with *Actinobacteria* (4) and *Proteobacteria* (2). *Novosphingobium* (negatively), *Quadrisphaera* (positively), and uncultured *Gemmatimonadaceae* (negatively) were detected both in NW and NS and showed the same trend with NO_3_^−^ concentration. Subgroup_6, Subgroup_7, Subgroup_12, Subgroup_22, RB41, JGI_0001001-H03, *Bryobacter*, *Candidatus*_Solibacter, and uncultured species belonged to *Acidobacteria* were negatively associated with NO_3_^−^ in NW. Besides three genera of *Thermoleophilia* class in the weathered rock, there were eight and four genera of *Actinobacteria* positively correlated with NO_3_^−^ in weathered rock and sediment, respectively. *Nitrospira* was only detected in NW with a negative correlation with NO_3_^−^ (Figure 4A). Species in *Gammaproteobacteria* and *Deltaproteobacteria* were mainly negatively associated with NO_3_^−^, while *Alphaproteobacteria* showed more diverse response to NO_3_^−^.

### 3.4. Bacterial interactions in weathered rocks and sediments

**Figure 5:**
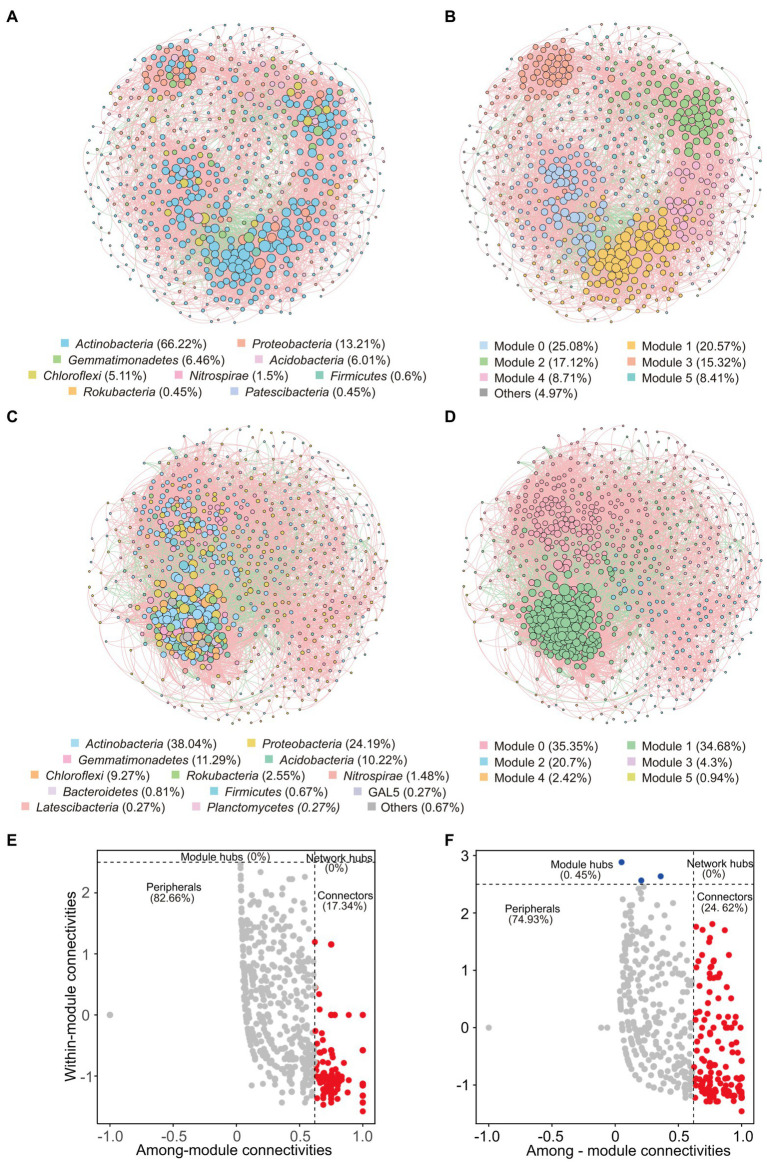
Co-occurrence networks of bacterial communities based on pairwise Spearman’s correlations between ASVs with a coefficient > |0.7| and a *p*-value < 0.01. The upper panel shows the network of weathered rocks with ASVs colored by taxonomy **(A)** and modularity **(B)**. The lower panel shows the network of sediments with ASVs colored by taxonomy **(C)** and modularity **(D)**. The size of each node is proportional to the number of connections. Red lines represent positive correlations and green lines represent negative correlations. Zi-Pi plots showing the distribution of ASVs with their topological roles in bacterial network of weathered rock **(E)** and sediment **(F)**.

Co-occurrence networks were conducted to reveal bacterial interactions. In total, there were 666 nodes (ASVs) and 8,217 edges in the co-occurrence network of weathered rocks (Co-NW; Figure 5A), whereas 744 nodes (ASVs) and 19,456 edges in the network for the sediment communities (Co-NS; Figure 5C). Most edges were positively linked (88.9% in Co-NW and 84.64% in Co-NS). Both networks showed good modularity with 6 main modules (Figures 5B,D). Nodes from the same or adjacent sampling sites tend to cluster in the same module. Topologically, Co-NW showed higher modularity (0.808), lower average clustering coefficient (0.549), higher average path length (3.511) than those in Co-NS (0.658, 0.553, 2.949; Table 2). Co-NW was more stable than Co-NS as estimated network stability by average degree.

**Table 2:** Topological indices of co-occurrence networks in the Chang Cave, Hubei province, central China.

Habitats	Co-occurrence networks							
	Nodes	Edges	AD	AWD	Diam	Modularity	ACC	APL
Weathered rock	666	8,217	24.676	31.127	13	0.808	0.549	3.511
Sediment	744	19,456	52.301	58.787	10	0.658	0.553	2.949

AD, average degree; AWD, average weighted degree; Diam, diameter; ACC, average clustering coefficient; APL, average path length.

Among the nodes, 129/164 keystone taxa were identified as connectors in Co-NW and Co-NS, respectively (Figures 5E,F). The module hubs were only identified in sediments (3 keystone taxa, accounted for 0.45%; Figure 5F). *Actinobacteria* predominated in all keystone taxa, which accounted for 68.99% and 34.73% of all keystone taxa in Co-NW and Co-NS, respectively. *Proteobacteria* ranked the second and accounted for 1.88% and 8.41% in Co-NW and Co-NS, respectively. One and three nodes affiliated with NC10 group were identified as connectors in Co-NW and Co-NS, respectively.

### 3.5. Potential functions of bacterial communities

**Figure 6:**
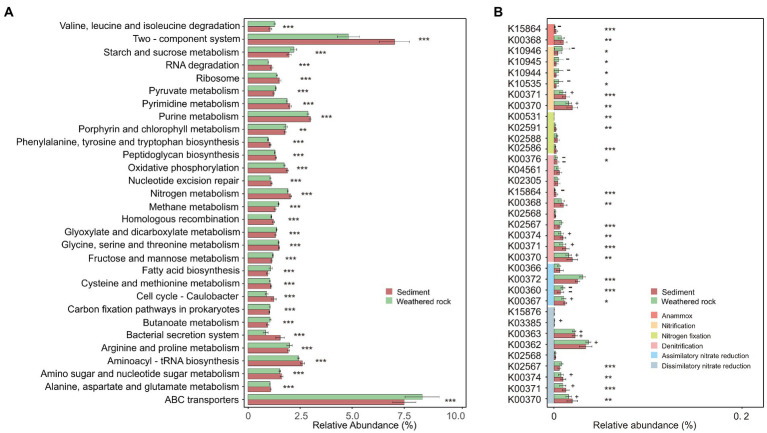
Relative abundance of the top 30 level-3 KEGG pathways **(A)** and nitrogen metabolism gene prediction **(B)** for bacterial communities in weathered rocks and sediments. Significance level: *p* < 0.05, *; *p* < 0.01, **; *p* < 0.001, ***. +/−: positively/negatively associated with nitrate.

Bacterial functions were predicted with Tax4Fun based on 16S rRNA gene sequence data. Results showed that bacterial functions in weathered rocks and sediments were significantly different as indicated by the relative abundances of top 30 level-3 KEGG pathways and Wilcoxon rank-sum tests (Figure 6A). “Nitrogen metabolism” showed a high abundance in sediments (*p* < 0.05), whereas “methane metabolism” and “prokaryotic carbon fixation pathways” were more abundant in weathered rock samples (*p* < 0.05; Figure 6A). It is worth mentioning that “nitrogen metabolism” with a relative abundance of 1.98% was more abundant than “methane metabolism” (1.38%) and “prokaryotic carbon fixation pathways” (1.03%). This observation was also supported by the results from FARPROTAX functional prediction, which confirmed the relatively high abundance of functions related to nitrogen cycling (Supplementary Figure S4).

KEGG functional genes related to “nitrogen metabolism” fell into six categories, which included dissimilatory/assimilatory nitrate reductions, denitrification, nitrogen fixation, nitrification, and anammox (Figure 6B). The nitrite reductase (NADH) [EC:1.7.1.15] had the highest relative abundance, followed by nitrate reductase [EC:1.7.5.1] and nitrite oxidoreductase [1.7.99.-]. The key genes responsible for nitrification and dissimilatory nitrate reduction were negatively and positively correlated with nitrate, respectively, in weathered rocks. Based on the results from FARPROTAX, weathered rocks had a higher abundance of nitrate reduction compared to those in sediments (3.90% and 0.68% in average, respectively). Sediments showed a higher abundance of aerobic ammonia oxidation and nitrification (7.66% and 8.90% in average, respectively) than those in weathered rocks (0.69% and 0.97% in average, respectively; Supplementary Figure S5).

## 4. Discussion

Following Europe and North America, China has the third largest acid rain region with acid rain mainly in the south of the Yangtze River (Shu et al., 2019). Despite the dominance of sulfate in acid rain, the ratio of SO_4_^2−^/NO_3_^−^ decreased by 81.9% in the period of 1998–2018, indicating the increase of NO_3_^−^ (Xuan et al., 2021). The annual average of wet nitrogen deposition increased from 11.11 to 13.87 kg ha^−1^ year^−1^ in China (Jia et al., 2014; Zhu et al., 2015). Dry nitrogen deposition mainly occurred in the North, East and Central China, and the average annual increase of dry deposition in the last decade was 1–2 kg N ha^−1^ year^−1^ (Jia et al., 2016). The Chang Cave is located in the acid rain area with high deposition of dry/wet nitrogen, receiving large amounts of exotic nitrate (Supplementary Figure S3; Jia et al., 2016; Yun et al., 2016a). The forest litter in overlying soil above the Chang Cave may also be a nitrate source (Hill, 1981), which can be transferred in to the cave by water along with organic-rich ammonia or ammonium to the dry passages (Pace, 1971; Hill, 1981; Hill et al., 1983). Previous studies have shown that rapid percolation of nitrate-rich water leads to elevated nitrate concentrations in the cavernous limestone (Knox and Moody, 1991; Boyer and Pasquarell, 1996). Other cave nitrate sources include bacterial nitrogen fixation (Faust, 1949, 1968; Lewis, 1992), bat guano (Hill, 1987), ammonium-urea from amberat (cave rat feces and urine; Moore and Sullivan, 1978), fertilizers, and sewage (Hess, 1900; Hill, 1981). Understanding of the impact of nitrate input on the microbial composition and functions in subsurface caves will help elucidate disturbance by human activities of ecological functions in the subsurface biosphere.

### 4.1. Nitrate impact differently on bacterial communities in weathered rocks and sediments

The application of nitrogen fertilizers is well-known to influence the structure of soil bacterial communities (Fierer et al., 2012a; Zhou et al., 2015). However, the effect of nitrate on microbial communities has often been neglected in previous studies related to karst caves. Nitrate input with acid rain or fertilization in overlying soils may result in the alteration of microbial diversity, microbial composition, and microbial functions in karst caves, which subsequently may have profound feedback on global climate change.

Nitrate contributed most to the distinguished bacterial communities in different habitats (Figures 2D–F) and resulted in unique indicator groups within each habitat. *Actinomyces, Crossiella*, and *Rubrobacteria* were indicator groups in weathered rocks, with *Betaproteobacteriales, Acidobacteria, Nitrosococcales*, *Nitrosococcaceae*, and *Nitrosomonadaceae* in sediments. Most indicator groups were related to nitrogen cycling. For example, the indicator phyla/order in weathered rock *Actinobacteria* is reported to be a major component of nitrate reducers in low-temperature peat and permafrost (Steven et al., 2008; Palmer et al., 2012; Palmer and Horn, 2012). *Crossiella*, an aerobic, non-motile actinomycete and a nitrate reducer, has already been described in subsurface environments (Portillo et al., 2009). In sediments *Betaproteobacteriales* are capable of denitrification, ammonia oxidation, nitrite oxidation, and nitrogen assimilation (Kalyuzhnaya et al., 2006). The subgroup_6 of *Acidobacteria* responds sensitively to high nitrogen availability in wetland soil (Hester et al., 2018). *Nitrosococcales*, *Nitrosococcaceae*, and *Nitrosomonadaceae* are ammonia-oxidizers (Ward et al., 2021). In addition, there were significant changes in the abundance of other bacteria due to the high input of exotic nitrate.

Besides the differences in indicator groups, bacterial communities also significantly differed in the alpha diversity and beta diversity between sediments and weathered rocks as indicated by alpha diversity indexes (Supplementary Figure S2) and NMDS analysis (Figure 2C). The alpha diversity of bacterial communities was higher in sediments than in the weathered rocks in the Chang Cave, consistent with observations in other caves (Ma et al., 2021; Ai et al., 2022). Moreover, our observation of the difference in the beta diversity between weathered rocks and sediments was also reported in other caves in Guilin city (Cao et al., 2021; Yang et al., 2021). These differences in bacterial diversities are likely results from the nitrate impact.

Nitrate may impact on bacterial alpha diversities *via* indirect or direct pathways in different habitats. In weathered rocks nitrate directly altered pH and thus indirectly impacted on bacterial diversity index (Figure 3A). In contrast, nitrate directly impacted bacterial diversity index in sediments (Figure 3B). Nitrate serves as a nutrient and electron acceptor in oligotrophic ecosystems (Spilde et al., 2005; Barton et al., 2010; Kimble et al., 2018). High nitrogen availability may reduce the abundance of nitrogen-fixing microbes (Berthrong et al., 2014) and stimulate nitrophilous taxa. Nitrifiers and denitrifiers which use inorganic N as energy sources or electron acceptors may competitively exclude other bacterial taxa (Campbell et al., 2010). Karst ecosystems based on chemoautotrophy are limited by the availability of inorganic energy sources (Fe, S, and N especially; Engel, 2007). Input of exogenous NO_3_^−^ increases the available nutrient content and may promote microbial development including but not limited to those involved in the nitrogen cycling. This presumption was supported by the significant positive correlation between bacterial alpha diversity and nitrate in the sediments (Figures 2B, 3B), a result from the direct impact of nitrate input. Nitrate input may decrease the pH, which primarily alters the composition of soil microbial communities (Zhang et al., 2014; Tian and Niu, 2015). Long-term N deposition at supersaturation decreases the microbial diversity due to soil acidification (Chung et al., 2007; Wang et al., 2018a,b), an indirect impact of nitrate on the bacterial diversity due to lowered pH.

Different from the soil systems, carbonates in karst caves showed strong buffer effect to nitrate input, which result in a slight drop in pH, thus favoring for cave microorganisms exposed to long-term alkaline stress. Bacterial diversity is the highest in neutral soils and decreases as soils become more alkaline (Fierer and Jackson, 2006). Alkaline conditions in karst caves may pose prolonged alkaline stress to microorganisms, and slight decrease in pH may benefit cave microbiota and lead to increasing bacterial diversity. The structural equation model analysis supported the decrease in the pH due to nitrate input, revealing a significant negative correlation between nitrate and pH in weathered rocks (Figures 2A, 3A). The negative correlation between the pH and alpha diversity confirmed that nitrate input increased the bacterial alpha diversity by lowering the pH (Figure 3A).

More genera significantly correlated with nitrate in the weathered rocks compared to sediments with the same criteria (Figure 4), suggesting stronger impact of nitrate on weathered rock. The input of nitrate had different effects on bacteria performing different functions or even a similar function. Except for *Lysobacter*, *Mesorhizobium*, and *Woeseia*, the bacteria in the N-cycle were negatively associated with nitrate, such as *Haliangium*, MND1, *Gaiella*, IS-44, Ellin6067, *Nitrospira*, *Nordella*, and *Novosphingobium*. *Woeseia*, IS-44, Ellin6067, and *Nitrospira* are involved in ammonia oxidation (Zhao et al., 2020; McCormick et al., 2021; Wang L. et al., 2021), and *Haliangium* is capable of nitrification–denitrification (Li et al., 2018). *Gaiella* and MND1 can reduce nitrate to nitrite (Albuquerque et al., 2011; McCormick et al., 2021). *Lysobacter*, *Mesorhizobium*, *Nordella*, and *Novosphingobium* are diazotrophic (Kaneko et al., 2000; Addison et al., 2007; Iwata et al., 2010; Cao et al., 2022). There are negative correlations between nitrate and bacteria involved in the Fe-Mn cycle, such as the iron-reducing bacteria *Acidibacter* and TRA3-20 and the manganese-oxidizing bacterium *Pedomicrobium* (Ridge et al., 2007; Nicomrat et al., 2008; Falagan and Johnson, 2014). *Constrictibacter* and *Stackebrandtia* that are responsible for cellulose decomposition and chitobiose degradation, respectively (Kang et al., 2012; Wang et al., 2019; Carrasco and Preston, 2020), showed significantly positive correlations with nitrate possibly resulting from alleviation of nitrogen limitation. Negative correlations between nitrate and *Acidobacteriia*, *Deltaproteobacteria*, *Gammaproteobacteria*, *Gemmatimonadetes*, and *Nitrospira* were also confirmed by Spearman’s tests, and *Actinobacteria* and *Chloroflexia* were positively associated with nitrate (Supplementary Table S1). Due to the positive and negative responses of microbial groups to nitrate concentration, nitrate input may subsequently alter their relative abundances as well as the bacterial composition and functions. Bacteria in weathered rocks were more impacted by nitrate due to higher contents of nitrate as opposed to those in sediments.

### 4.2. Bacterial interactions and functions

Consistent with previous studies (Cao et al., 2021; Ma et al., 2021; Yang et al., 2021), nodes in the co-occurrence network of weathered rocks and sediments were mostly positively linked (88.9% and 84.64%, respectively; Figures 5A–D), which suggested a prevalence of collaboration rather than competition to overcome nutrient limitation in oligotrophic caves. The higher modularity of Co-NW indicated that bacterial populations form closer collective structures, in which they mainly cooperated and established mutualistic relationships to maintain the stability of the ecosystem. More connectors were detected in Co-NW than in Co-NS, but module hubs were only detected in the sediments, suggesting a less hub-based and more connected structure in the weathered rocks.

Keystone taxa in networks are considered to play critical ecological roles to sustain the health of the bacterial ecosystems. Most keystone taxa in this study were involved in nitrogen cycling, indicating the fundamental role of nitrogen in subsurface karst ecosystems (Supplementary Table S2). For example, the NC10 group as connectors in this work and previous studies (Cheng et al., 2021b; Yang et al., 2021) has been demonstrated to oxidize CH_4_ anaerobically coupled with nitrite reduction (Ettwig et al., 2008). This is a clear example the coupling of carbon and nitrogen cycling in the subsurface biosphere. Moreover, this also suggested a potential contribution by NC10 to anaerobic methane oxidation besides aerobic consumption of methane by the Upland Soil Cluster (Cheng et al., 2021a). Wb1-P19 is a genus named after an uncultured clone in cave water, which phylogenetically clusters with sulfur- or nitrite-oxidizing autotrophs (Holmes et al., 2001). Species in *Conexibacter*, *Sphingomonas*, *Kribbella*, *Nocardioides*, *Amycolatopsis*, *Streptomyces*, *Actinophytocola*, *Aeromicrobium*, *Stackebrandtia*, and *Rubrobacter* can reduce nitrate into nitrite (Pranamuda et al., 1997; Monciardini et al., 2003; Keulen et al., 2005; Labeda and Kroppenstedt, 2005; Prakash and Lal, 2006; Ramasamy et al., 2012; Seki et al., 2012; Suzuki, 2015; Bouznada et al., 2016; Guerrero-Cruz et al., 2019; Curtis et al., 2020). *Nitrosospira* is a ubiquitous ammonium-oxidizing bacterium (AOB) found in various environments (Norton et al., 2008). The comammox *Nitrospira*, capable of oxidizing ammonia completely to nitrate (Daims et al., 2015; van Kessel et al., 2015) was recently reported in the Heshang Cave (Ma et al., 2021) and served as connector in Co-NW in the Chang Cave. This expands our knowledge about the distribution of comammox in natural environments. Species in *Gemmatimonas* were found to reduce N_2_O (Chee-Sanford et al., 2019). Denitrification and dissimilatory nitrate reduction activities have been observed in *Bacillus* species (Heylen and Keltjens, 2012). *Azotobacter paspali* lacks the nitrate reductase enzyme and is able to fix nitrogen in the presence of nitrate (Stephan et al., 1997). The clade RB41 is a group of rhizospheric bacteria, some possibly assigned to *Acidobacteria*, that promote host plant uptake of nitrogen (Fu et al., 2021). Subgroup_2, Subgroup_6, Subgroup_7, Subgroup_13, and *Solirubrobacter* are thought to be related to nitrogen transformations (Liu et al., 2017; Gao et al., 2022). Genomic information indicated that *Dyella* has the potential to perform nitrate reduction (Too et al., 2018), and Ga0077536 is involved in nitrogen fixation (Gonzalez-Pimentel et al., 2021). 67–14 in the order of *Solirubrobacterales* is reported to be associated with autotrophic CO_2_ fixation and ferrous iron redox reactions (Rodriguez et al., 2022), commonly found in environments with limited concentrations of TOC (Filippini et al., 2019).

Consistent with the functions of keystone taxa in the co-occurrence networks, high relative gene abundances of nitrogen metabolism were indicated by Tax4Fun2, with the dominance of dissimilatory and assimilatory nitrate reductions (Figures 6A,B). Heatmap analysis also showed the significant enrichment for processes involved in the nitrogen cycle (Supplementary Figure S4). Nitrification is an important pathway in various ecosystems, but the input of exogenous nitrate in weathered rocks may have inhibited nitrification (Figure 6B), reducing the production of N_2_O as a by-product (Hirsch and Mauchline, 2015). Dissimilatory nitrate reduction to ammonium (DNRA) converts NO_3_^−^ to NH_4_^+^, thus providing additional NH_4_^+^ to primary producers (Rütting et al., 2011; Minick et al., 2016; Pandey et al., 2020). DNRA can be coupled with the oxidation of various electron donors such as organic matter, methane, sulfur compounds, H_2_ or iron (Zumft, 1997; Cardoso et al., 2006; Weber et al., 2006; Haroon et al., 2013; Ettwig et al., 2016). The highest relative abundance of DNRA was found in weathered rocks in our study (Figure 6B). The assimilatory nitrate reduction process reduces NO_3_^−^ to NH_4_^+^ by the nitrate reductase and assimilatory nitrite reductase with NADH as the reducing power. Nitrate reduction was more commonly observed in weathered rocks, whereas aerobic ammonia oxidation and nitrification were prevalent in the sediments (Supplementary Figure S5).

Besides the genes related to N-metabolism, high abundance of genes involved in methane metabolism and carbon fixation were also detected, indicating ecological roles in C_1_-cycling of cave microorganisms. Methane oxidizing bacteria, particularly the Upland Soil Cluster, responsible for the oxidation of trace levels of methane in the air, have been reported to be widely distributed in caves (Zhao et al., 2018; Cheng et al., 2021a), thus coinciding with our results of function prediction. In addition to USC, ASVs affiliated NC10 capable of coupling anaerobic oxidation of methane with nitrite reduction may also contribute to C_1_ and N cycles in the cave. This provides another clue to investigate the ecological function of cave as methane sink, and merits further studies. Genes involved in methane oxidation were most likely inhibited by exogenous nitrate input in the cave, especially in the weathered rock (Supplementary Table S4). Furthermore, negative correlations between nitrate concentration and the abundance of *Gammaproteobacteria* (including low-affinity methanotrophs) and NC10 were found (Mills et al., 2013; Webster et al., 2022; Supplementary Table S4). High-affinity methanotrophs, known as the Upland Soil Cluster (USC) α and γ clades, may be responsible for atmospheric-CH_4_ consumption in caves (Zhao et al., 2018; Cheng et al., 2021a), and their response to nitrate input is not known. Further study is needed to determine the specific effect of nitrate input on cave methane fluxes. Carbon fixation pathways were dominated by the reductive acetyl-CoA pathway (Wood-Ljungdahl pathway) and 3-hydroxypropionate bicycle (Supplementary Figure S6). The reductive acetyl-CoA pathway conserves energy from CO_2_ or C_1_ compounds, and is considered to be the primary route of CO_2_ fixation in Lava caves (Selensky et al., 2021). In karst caves, the high concentrations of CO_2_ and HCO_3_^−^ and limited amount of organic matter derived from photosynthesis favor C_1_ fixation *via* the Calvin-Benson cycle. The 3-hydroxypropionate bicycle has been found in *Chloroflexus* (Hugler et al., 2002; Lavoie et al., 2017). Key enzymes were identified involving in reductive pentose phosphate cycle (Calvin cycle), reductive tricarboxylic acid (TCA) cycle, reductive acetyl-CoA pathway (Wood-Ljungdahl pathway), 3-hydroxypropionate bicycle, 3-hydroxypropionate/4-hydroxybutylate cycle, and dicarboxylate/4-hydroxybutyrate cycle (Supplementary Table S3). The absence of propionyl-CoA synthase in 3-hydroxypropionate bicycle serves as additional evidence to support autotrophic bacteria as the main primary producers in karst caves (Cañveras et al., 2001; Parker et al., 2013; Jones and Macalady, 2016; Dong et al., 2020). Nitrate was positively correlated with genes responsible for the Calvin-Benson cycle (Supplementary Table S3), employed by numerous groups of autotrophic bacteria such as H_2_-, Fe^2+^-, S-, and NH_4_^+^-oxidizers (Raven, 2009; Claassens et al., 2018). In contrast, nitrate was negatively correlated with the TCA cycle (Supplementary Table S3). The 3-hydroxypropionate bicycle genes were positively associated with nitrate in carbon fixation pathways in the Chang Cave, consistent with *Chloroflexia* abundance being enhanced by nitrate input (Supplementary Table S1). In contrast, genes of the reductive citrate cycle (Arnon-Buchanan cycle) were negatively associated with nitrate in the Chang cave (Supplementary Table S4). To be note, partially sequenced 16S rRNA genes fail to distinguish taxa beyond the genus level (Heidrich and Beule, 2022), and functional prediction accuracy depends on the size and research area of the reference gene database (Mongad et al., 2021). Uncertainty dose exist in functional predictions based on the partially sequenced 16S rRNA gene sequence data (Mongad et al., 2021; Heidrich and Beule, 2022), which can be overcome *via* metagenome sequencing in near future.

## 5. Conclusion

This study revealed highly diverse bacterial communities in the Chang Cave with strong habitat specialization. For the first time, we demonstrated the impact of nitrate on the composition, diversity, interaction and function of bacterial communities in karst caves. Nitrate shaped bacterial communities across different habitats with an explanation of 27.2%. It increased the bacterial alpha diversity directly in the sediments and indirectly in weathered rocks *via* lowering the pH. Nitrate also directly increased the beta diversity of bacterial communities, resulting in high habitat specialization. More genera were significantly correlated with nitrate in the weathered rocks than in the sediment samples, suggesting a strong impact of nitrate on weathered rocks. Keystone taxa involved in nitrogen cycling were detected such as *Rokubacteriales* belonging to NC10, capable of nitrite reduction coupling with anaerobic methane oxidation, and comammox *Nitrospira,* completely oxidizing ammonia to nitrate, and various nitrate-reducers. Results of function prediction also confirmed the dominance of the genes related to nitrogen metabolism followed by those in methane metabolism and carbon fixation. Elevated nitrate concentration may also have shifted the pathway of carbon fixation in caves, enhancing the Calvin-Benson cycle and 3-hydroxypropionate bicycle, but inhibiting the reductive tricarboxylic acid and reductive citrate cycles. The results enhance our understanding on the N-cycling in karst caves and offer a new window to study the impact of anthropogenic activities on subsurface biosphere and ecological functions of caves in terms of nitrogen and carbon cycling under the context of global change.

## Data availability statement

The datasets presented in this study can be found in online repositories. The names of the repository/repositories and accession number(s) can be found at: https://www.ncbi.nlm.nih.gov/, SRR22513012–SRR22513071.

## Author contributions

XLiu, HW, and WW contributed to conception and design of the study. XLiu organized the database and performed the statistical analysis. WW, XC, and YW helped data mining. XLiu wrote the first draft of the manuscript. LL, QL, LM, and XLu wrote sections of the manuscript. HW and OT finalized the manuscript. All authors contributed to the manuscript revision, read, and approved the submitted version.

## Funding

This work was supported by National Natural Science Foundation of China (no. 91951208).

## Conflict of interest

The authors declare that the research was conducted in the absence of any commercial or financial relationships that could be construed as a potential conflict of interest.

The handling editor YS declared a shared affiliation with the authors XLiu, HW, WW, XC, YW, QL, LL, LM, and XLu at the time of review.

## Publisher’s note

All claims expressed in this article are solely those of the authors and do not necessarily represent those of their affiliated organizations, or those of the publisher, the editors and the reviewers. Any product that may be evaluated in this article, or claim that may be made by its manufacturer, is not guaranteed or endorsed by the publisher.

## Footnotes

^1^ http://huttenhower.sph.harvard.edu/galaxy
