# Supplementary material for: Nitrate determines the bacterial habitat specialization and impacts microbial functions in a subsurface karst cave
Source: Front Microbiol. 2023 Feb 9;14:1115449. doi: 10.3389/fmicb.2023.1115449 (PMC9947541; doi:10.3389/fmicb.2023.1115449)
Supplement: Supplementary file 1 [file Data_Sheet_1.pdf]

Supplementary Material

Nitrate determines the bacterial niche specificity and impacts microbial functions in a subsurface karst cave environment

Xiaoyan Liu, Hongmei Wanga*, Weiqi Wang, Xiaoyu Cheng, Yiheng Wang, Qing Li, Lu Li, Liyuan Ma, Xiaolu Lu, Olli H Tuovinen

*** Correspondence:** Prof. Hongmei Wang: wanghmei04@163.com or hmwang@cug.edu.cn

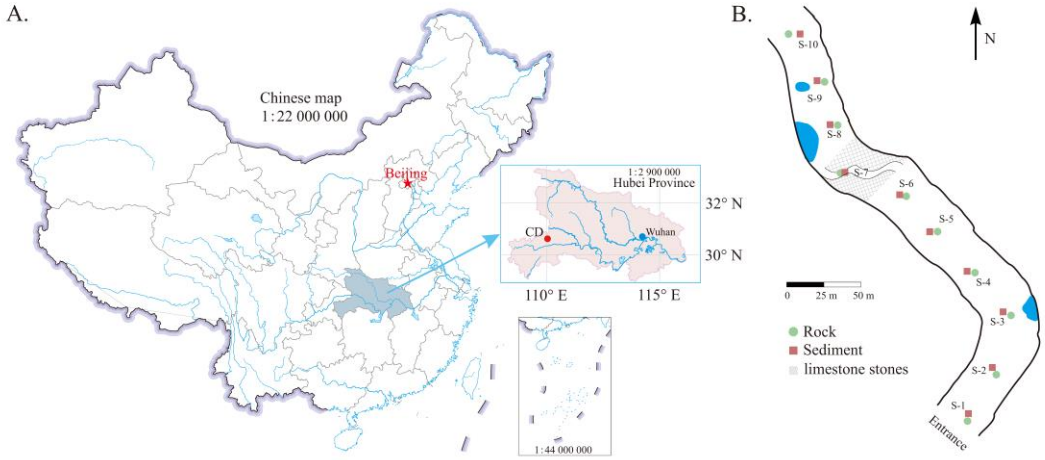

Supplementary Figure 1. The geographic location (30°39′26.01″N, 109°58′27.49″ E) (A) and schematic diagram of the sampling site in the Chang Cave (B), Hubei province.

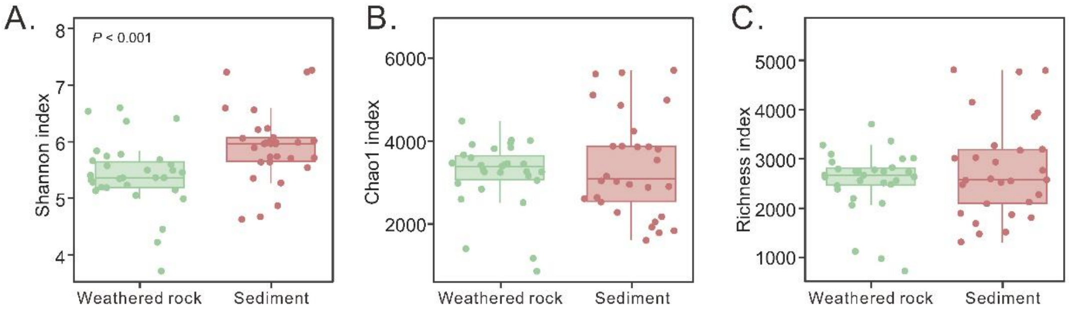

Supplementary Figure 2. The alpha diversity (Shannon index (A), Chao1 index (B) and Richness index (C)) of weathered rocks and sediments in the Chang Cave.

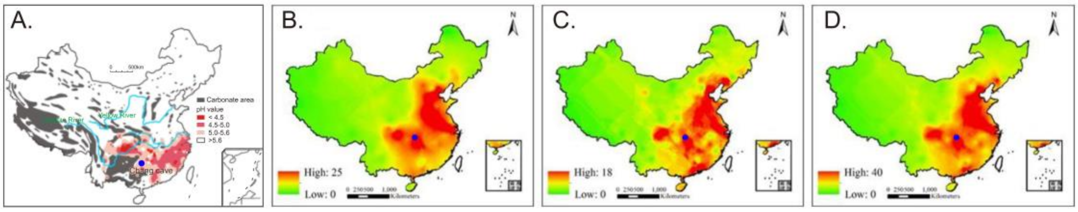

Supplementary Figure 3. (A) Location of the Chang Cave on acid rain distribution map in China, modified after http://english.mep.gov.cn/standards_reports/soe/soe2011/201307/t20130712_255427.htm, Yuan and Cao (2008), and Yun et al. (2016). (B-D) Location of the Chang Cave in the map of spatial distribution characteristics of atmospheric inorganic nitrogen wet deposition in China, 2011-2015, modified after Jia et al. (2019). Data exclude Taiwan and the South China Sea islands of China. (B) NH_4^+-N; (C) NO_3^--N; (D) NH_4^+-N + NO_3^--N. Unit in kg N ha^{-1} yr^{-1}. The blue dot in the map shows the location of the Chang Cave.

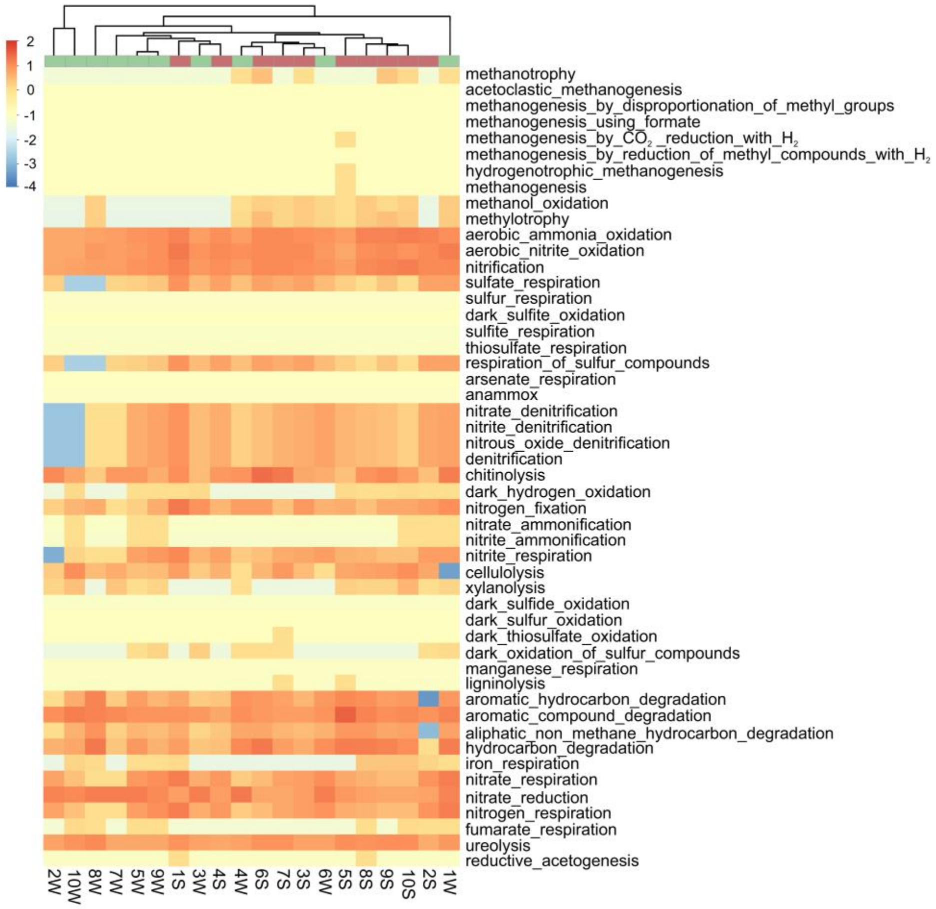

Supplementary Figure 4. Heatmap of the top 50 functions predicted by FARPROTAX related to biochemical cycle processes.

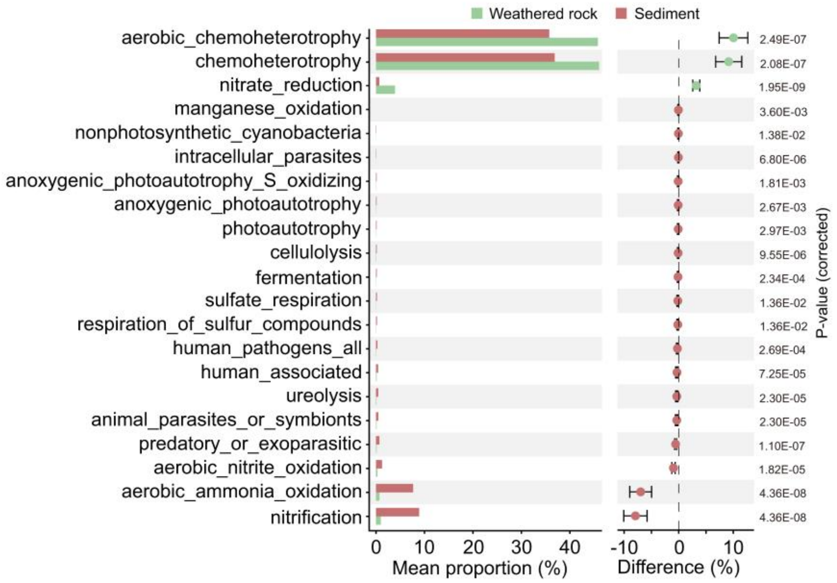

Supplementary Figure 5. Predicted functions of biochemical cycle processes in weathered rocks and sediments by FARPROTAX. Only the top 21 functions with significant differences between weathered rocks and sediments, and the relative abundance > 0.3% were selected.

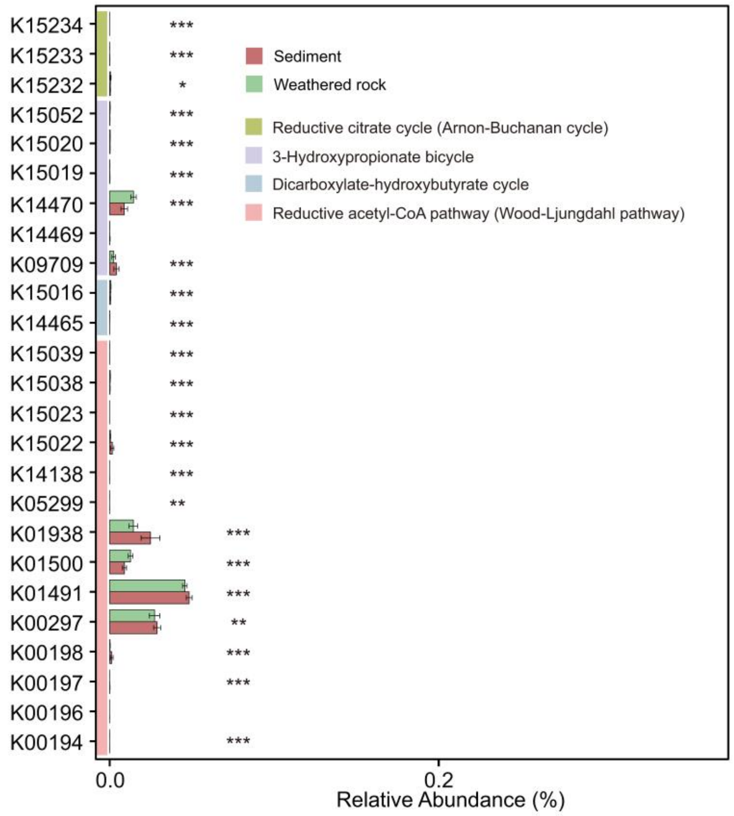

Supplementary Figure 6. Relative abundance of the predicted genes related to prokaryotic carbon fixation in weathered rocks and sediments. Significance level: $P < 0.05$, *; $P < 0.01$, **; $P < 0.001$, ***.

Supplementary Table 1. Microbial classes significantly associated with nitrate with a Spearman rank correlation coefficient < 0.05.

Class	Weathered rock	Sediment
ABY1	-0.49	/
Acidobacteriia	-0.62	/
Actinobacteria	0.48	/
AKAU4049	0.39	/
Anaerolineae	-0.45	0.41
AT-s3-28	/	0.47
BD7-11	-0.38	/
*Blastocatellia*_(Subgroup_4)	-0.57	/
BRC1	-0.49	/
Campylobacteria	-0.54	/
Chloroflexia	0.45	/
Chthonomonadetes	-0.46	-0.49
Deferribacteres	-0.42	/
Deltaproteobacteria	-0.55	/
FFCH5909	-0.39	/
Gammaproteobacteria	-0.47	/
Gemmatimonadetes	-0.38	-0.53
Gitt-GS-136	0.49	/
Holophagae	-0.65	-0.39
Latescibacteria	-0.4	/
LD1-PA32	/	0.43
Lineage_IIa	-0.53	/
Lineage_IIb	-0.37	/

Longimicrobia	/	0.47
MB-A2-108	/	-0.42
NC10	-0.42	/
Nitrospira	-0.55	/
OM190	-0.47	/
PAUC43f_marine_benthic_group	0.38	0.37
Planctomycetacia	-0.52	/
Rhodothermia	/	0.56
S0134_terrestrial_group	0.44	/
Subgroup_11	-0.43	/
Subgroup_17	-0.45	/
Subgroup_18	-0.55	/
Subgroup_21	/	0.48
Subgroup_22	-0.55	/
Subgroup_25	-0.45	/
Subgroup_5	-0.39	/
Subgroup_6	-0.68	/
Thermoleophilia	-0.47	/
TK10	-0.6	/
unclassified_*Acidobacteria*	-0.55	0.45
unclassified_*Actinobacteria*	/	-0.38
unclassified_*Gemmatimonadetes*	0.6	/
unculture	-0.39	-0.38
Verrucomicrobiae	-0.5	/
WS6_(Dojkabacteria)	/	0.51
Zixibacteria	-0.57	/

/: no significant correlation.

Supplementary Table 2. Detailed affiliations of the keystone ASVs in the Zi-Pi plots. Bolded species are reported to be involved in nitrogen metabolism previously.

Network	Classification	No. of ASVs	Phylum	Genus
Weathered Rock	Connector	8	*Acidobacteria*	**Subgroup_7 (2)**, **Subgroup_6 (2)**, Subgroup_10, **RB41**, JGI_0001001-H03, uncultured
		3	*Chloroflexi*	*Thermobaculum*, AKYG1722, JG30-KF-CM66
		1	*Firmicutes*	***Bacillus***
		8	*Gemmatimonadetes*	***Gemmatimonas***, unclassified_*Gemmatimonadaceae*, uncultured (6)
		4	*Nitrospirae*	***Nitrospira* (4)**
		1	*Patescibacteria*	*Saccharimonadales*
		14	*Proteobacteria*	***Nitrosospira* (3)**, **MND1 (2)**, ***Sphingomonas* (2)**, uncultured (2), *Dongia*, *Woeseia*, *Escherichia-Shigella*, unclassified_*Rhizobiaceae*, unclassified_*Hyphomicrobiaceae*
		1	*Rokubacteria*	***Rokubacteriales***

		89	*Actinobacteria*	0319-7L14, 67-14 (12), ***Amycolatopsis***, ***Conexibacter* (2)**, ***Crossiella* (13)**, ***Gaiella* (4)**, *Haloactinopolyspora*, IMCC26256 (2), ***Kribbella* (4)**, MB-A2-108, ***Nocardioides***, *Promicromonospora*, ***Pseudonocardia* (12)**, ***Rubrobacter* (10)**, ***Solirubrobacter* (3)**, *Sporichthya*, ***Stackebrandtia***, ***Streptomyces***, unclassified_*Acidimicrobiia*, unclassified_*Actinobacteria*(4), unclassified_*Frankiales*, unclassified_*Nocardioidaceae*, unclassified_*Pseudonocardiaceae*, uncultured(10)
Sediment	Module hub	2	Proteobacteria	**wb1-P19**, **IS-44**
		1	Chloroflexi	uncultured
	Connector	15	*Acidobacteria*	*Bryobacter*, **RB41 (2)**, Subgroup_10 (3), **Subgroup_2**, Subgroup_20, Subgroup_22, **Subgroup_6 (4)**, Subgroup_9
		58	*Actinobacteria*	0319-7L14 (2), 67-14 (4), ***Actinophytocola***, ***Aeromicrobium***, ***Amycolatopsis***, ***Crossiella* (10)**, ***Gaiella* (3)**, IMCC26256 (8), MB-A2-108 (3), ***Nocardioides* (2)**, ***Pseudonocardia* (2)**, *Quadrisphaera*, ***Streptomyces***, unclassified_*Actinobacteria*, unclassified_*Propionibacteriaceae*, uncultured (17)
		18	***Chloroflexi***	AKYG1722, Gitt-GS-136 (6), JG30-KF-CM45 (2), JG30-KF-CM66 (2), KD4-96 (5), TK10 (2)
		2	*Firmicutes*	***Bacillus* (2)**
		1	*GAL15*	GAL15
		11	***Gemmatimonadetes***	unclassified_*Gemmatimonadaceae* (2), uncultured (9)
		2	*Latescibacteria*	***Latescibacteria***

54	*Proteobacteria*	*Acetobacter*, bacteriap25, *Dongia* (2), ***Dyella***, **Ellin6055**, **Ga0077536 (2)**, ***Haliangium***, **IS-44 (2)**, **MND1 (8)**, **NB1-j**, *Nordella*, ***Novosphingobium***, PLTA13 (2), ***Sphingomonas***, ***Steroidobacter***, **SWB02**, TRA3-20, unclassified_*Hyphomicrobiaceae*, unclassified_*Methyloligellaceae*, unclassified_*Rhizobiaceae*, unclassified_*Sphingomonadaceae*, uncultured (11), **wb1-P19 (9)**, ***Woeseia***
3	*Rokubacteria*	***Rokubacteriales* (3)**

Supplementary Table 3. The relative abundance of key functional genes in the six microbial carbon fixation pathways.

Microbial carbon fixtion pathways	gene	Group	RPM	tax
Calvin cycle	K00314	Weathered rock (+)	1.91E-05	sarcosine dehydrogenase [EC:1.5.8.3]
		Sediment	2.10E-05	
	**K00150**	Weathered rock	3.70E-06	glyceraldehyde-3-phosphate dehydrogenase (NAD(P)) [EC:1.2.1.59]
		Sediment	2.90E-06	
	**K00615**	Weathered rock	1.27E-03	transketolase [EC:2.2.1.1]
		Sediment	1.41E-03	
	**K00855**	Weathered rock	1.83E-05	phosphoribulokinase [EC:2.7.1.19]
		Sediment	5.65E-05	
	**K00927**	Weathered rock	5.94E-04	phosphoribulokinase [EC:2.7.1.19]
		Sediment	6.64E-04	
	**K01601**	Weathered rock	4.41E-05	ribulose-bisphosphate carboxylase large chain [EC:4.1.1.39]
		Sediment	1.27E-04	

**K01602**	Weathered rock	6.84E-06	ribulose-bisphosphate carboxylase small chain [EC:4.1.1.39]
	Sediment	1.85E-05	
**K01623**	Weathered rock (+)	7.20E-05	fructose-bisphosphate aldolase, class I [EC:4.1.2.13]
	Sediment	8.09E-05	
**K01624**	Weathered rock (+)	4.89E-04	fructose-bisphosphate aldolase, class II [EC:4.1.2.13]
	Sediment	4.33E-04	
**K01807**	Weathered rock (-)	3.30E-05	ribose 5-phosphate isomerase A [EC:5.3.1.6]
	Sediment	1.17E-04	
**K01808**	Weathered rock	3.22E-04	ribose 5-phosphate isomerase B [EC:5.3.1.6]
	Sediment	2.53E-04	
**K02446**	Weathered rock (+)	4.21E-04	fructose-1,6-bisphosphatase II [EC:3.1.3.11]
	Sediment	2.63E-04	
**K03841**	Weathered rock (-)	6.75E-05	fructose-1,6-bisphosphatase I [EC:3.1.3.11]
	Sediment	2.31E-04	
**K11532**	Weathered rock	2.04E-11	fructose-1,6-bisphosphatase II / sedoheptulose-1,7-bisphosphatase

		Sediment	1.40E-08	[EC:3.1.3.11 3.1.3.37]
	K00169	Weathered rock	5.80E-05	pyruvate ferredoxin oxidoreductase, alpha subunit [EC:1.2.7.1]
		Sediment	7.37E-05	
	K00170	Weathered rock (-)	6.39E-05	pyruvate ferredoxin oxidoreductase, beta subunit [EC:1.2.7.1]
		Sediment	7.83E-05	
	K00171	Weathered rock (-)	6.39E-06	pyruvate ferredoxin oxidoreductase, delta subunit [EC:1.2.7.1]
		Sediment	6.00E-06	
Reductive tricarboxylic acid cycle	K00172	Weathered rock (-)	1.72E-05	pyruvate ferredoxin oxidoreductase, gamma subunit [EC:1.2.7.1]
		Sediment	2.71E-05	
	K00174	Weathered rock	7.29E-04	2-oxoglutarate ferredoxin oxidoreductase subunit alpha [EC:1.2.7.3]
		Sediment	6.65E-04	
	K00175	Weathered rock	4.08E-04	2-oxoglutarate ferredoxin oxidoreductase subunit beta [EC:1.2.7.3]
		Sediment	3.87E-04	
	K00176	Weathered rock (-)	3.02E-06	2-oxoglutarate ferredoxin oxidoreductase subunit delta [EC:1.2.7.3]

		Sediment	1.17E-05	
	**K00177**	Weathered rock (-)	4.37E-06	2-oxoglutarate ferredoxin oxidoreductase subunit gamma [EC:1.2.7.3]
		Sediment	1.76E-05	
	**K01637**	Weathered rock (+)	7.09E-04	isocitrate lyase [EC:4.1.3.1]
		Sediment	4.74E-04	
	**K01643**	Weathered rock (-)	7.07E-06	citrate lyase subunit alpha / citrate CoA-transferase [EC:4.1.3.6 2.8.3.10]
		Sediment	2.75E-05	
	**K01644**	Weathered rock (+)	7.33E-04	citrate lyase subunit beta / citryl-CoA lyase [EC:4.1.3.6 4.1.3.34]
		Sediment	5.20E-04	
	**K01646**	Weathered rock (-)	2.40E-07	citrate lyase subunit gamma [EC:4.1.3.6]
		Sediment	1.06E-06	
3-Hydroxypropionate bicycle (key enzyme not detected: Propionyl-CoA synthase)	K14468	Weathered rock	3.59E-07	malonyl-CoA reductase / 3-hydroxypropionate dehydrogenase (NADP+) [EC:1.2.1.75 1.1.1.298]
		Sediment	6.44E-07	
	**K08691**	Weathered rock (-)	8.39E-05	malyl-CoA lyase [EC:4.1.3.24]
		Sediment	6.22E-05	

	K14450	Weathered rock	1.83E-07	L-malyl-CoA/beta-methylmalyl-CoA lyase
		Sediment (+)	1.92E-07	
	**K08691**	Weathered rock (-)	8.39E-05	malyl-CoA lyase [EC:4.1.3.24]
		Sediment	6.22E-05	
	**K11263**	Weathered rock (+)	1.22E-03	acetyl-/propionyl-CoA carboxylase, biotin carboxylase, biotin carboxyl carrier protein [EC:6.3.4.14]
		Sediment	6.74E-04	
	K15036	Weathered rock	4.09E-06	acetyl-CoA/propionyl-CoA carboxylase [EC:6.4.1.2 6.4.1.3]
		Sediment	4.73E-06	
3-Hydroxypropionate/4-hydroxybutyrate cycle	**K01964**	Weathered rock (+)	2.77E-09	acetyl-CoA/propionyl-CoA carboxylase [EC:6.4.1.2 6.4.1.3]
		Sediment (+)	7.99E-09	
	**K14534**	Weathered rock	9.93E-07	4-hydroxybutyryl-CoA dehydratase / vinylacetyl-CoA-Delta-isomerase [EC:4.2.1.120 5.3.3.3]
		Sediment	2.14E-06	
	K14468	Weathered rock	3.59E-07	malonyl-CoA reductase / 3-hydroxypropionate dehydrogenase (NADP+) [EC:1.2.1.75 1.1.1.298]
		Sediment	6.44E-07	

Dicarboxylate/ 4-hydroxybutyrate cycle	**K01595**	Weathered rock (-)	8.40E-04	phosphoenolpyruvate carboxylase [EC:4.1.1.31]
		Sediment (-)	1.01E-03	
	**K15016**	Weathered rock (+)	7.49E-06	3-hydroxybutyryl-CoA dehydratase / 3-hydroxyacyl-CoA dehydrogenase [EC:4.2.1.55 1.1.1.35]
		Sediment (+)	5.01E-06	
	**K14465**	Weathered rock	9.18E-07	succinate semialdehyde reductase (NADPH) [EC:1.1.1.-]
		Sediment	3.20E-07	
Reductive acetyl-CoA pathway (Wood-Ljungdahl pathway)	**K00190**	Weathered rock	1.40E-10	carbon monoxide dehydrogenase / acetyl-CoA synthase subunit beta [EC:1.2.7.4 1.2.99.2 2.3.1.169]
		Sediment	6.88E-09	
	**K14138**	Weathered rock	2.32E-09	carbon monoxide dehydrogenase / acetyl-CoA synthase subunit alpha [EC:1.2.7.4 1.2.99.2 2.3.1.169]
		Sediment	3.45E-08	

The genes with significant differences ($P < 0.05$) between weathered rocks and sediments are shown in bold. +/-: positively/ negatively correlated with nitrate.

Supplementary Table 4. Key enzymes involved in methane metabolism and carbon fixation pathways in prokaryotes with significant correlation with NO_3^- in weathered rocks and sediments. Significant Spearman rank correlation coefficients (*P* < 0.05) were shown.

	KO	KO3	KO4	EC	WR	S
Methane oxidation, methanotroph	K14028	Methane metabolism	mdh1, mxaF; methanol dehydrogenase (cytochrome c) subunit 1	[EC:1.1.2.7]	-0.46	/
	K14029		mdh2, mxaI; methanol dehydrogenase (cytochrome c) subunit 2	[EC:1.1.2.7]	-0.55	/
	K16254		mxaJ; mxaJ protein	[EC:1.1.2.7]	-0.56	/
	K16255		mxaG; cytochrome c-L	[EC:1.1.2.7]	-0.6	/
	K16256		mxaA; mxaA protein	[EC:1.1.2.7]	-0.58	/
	K16257		mxaC; mxaC protein	[EC:1.1.2.7]	-0.6	/
	K16258		mxaK; mxaK protein	[EC:1.1.2.7]	-0.6	/
	K16259		mxaL; mxaL protein	[EC:1.1.2.7]	-0.6	/
	K16260		mxaD; mxaD protein	[EC:1.1.2.7]	-0.62	/

Phosphate acetyltransferase-acetate kinase pathway	K13788		pta; phosphate acetyltransferase	[EC:2.3.1.8]	0.39	/
	K08691		mcl; malyl-CoA/(S)-citramalyl-CoA lyase	[EC:4.1.3.24 4.1.3.25]	-0.47	/
	K14449		mch, mcd; 2-methylfumaryl-CoA hydratase	[EC:4.2.1.148]	-0.44	/
	K14472		smtB; succinyl-CoA:(S)-malate CoA-transferase subunit B	[EC:2.8.3.22]	0.44	/
3-Hydroxypropionate bicycle	K15018	Carbon fixation pathways in prokaryotes	3-hydroxypropionyl-coenzyme A synthetase	[EC:6.2.1.36]	0.38	/
	K15019		3-hydroxypropionyl-coenzyme A dehydratase	[EC:4.2.1.116]	0.37	/
	K00240		sdhB, frdB; succinate dehydrogenase / fumarate reductase, iron-sulfur subunit	[EC:1.3.5.1 1.3.5.4]	/	0.37
	K01964		acetyl-CoA/propionyl-CoA carboxylase	[EC:6.4.1.2 6.4.1.3]	/	0.43
	K18594		3-hydroxypropionyl-CoA synthetase (ADP-forming)	[EC:6.2.1.-]	/	0.41
	K18605		biotin carboxyl carrier protein	[EC:6.4.1.2]	/	-0.49
Dicarboxylate-hydroxybutyra	K14534		abfD; 4-hydroxybutyryl-CoA dehydratase / vinylacetyl-CoA-Delta-isomerase	[EC:4.2.1.120 5.3.3.3]	-0.57	/

te cycle	K18593	4-hydroxybutyryl-CoA synthetase (ADP-forming)	[EC:6.2.1.-]	/	0.51
	K18605	biotin carboxyl carrier protein	[EC:6.4.1.2]	-0.42	/
Hydroxypropionate-hydroxybutyrate cycle	K15020	acryloyl-coenzyme A reductase	[EC:1.3.1.84]	/	0.49
	K15036	acetyl-CoA/propionyl-CoA carboxylase	[EC:6.4.1.2 6.4.1.3 2.1.3.15]	/	0.44
	K15052	propionyl-CoA carboxylase	[EC:6.4.1.3 2.1.3.15]	/	0.44
Incomplete reductive citrate cycle	K18209	tfrA; fumarate reductase (CoM/CoB) subunit A	[EC:1.3.4.1]	-0.38	/
Phosphate acetyltransferase-acetate kinase pathway	K00925	ackA; acetate kinase	[EC:2.7.2.1]	/	-0.47
Reductive acetyl-CoA pathway (Wood-Ljungdahl pathway)	K00196	cooF; anaerobic carbon-monoxide dehydrogenase iron sulfur subunit	[EC:1.2.7.4]	-0.51	/
	K01938	fhs; formate--tetrahydrofolate ligase	[EC:6.3.4.3]	-0.51	/
Reductive	K00031	IDH1, IDH2, icd; isocitrate dehydrogenase	[EC:1.1.1.42]	-	/

citrate cycle (Arnon-Buchanan cycle)				0.42	
	K01595	ppc; phosphoenolpyruvate carboxylase	[EC:4.1.1.31]	-0.37	/
	K01681	ACO, acnA; aconitate hydratase	[EC:4.2.1.3]	-0.37	/
	K01903	sucC; succinyl-CoA synthetase beta subunit	[EC:6.2.1.5]	-0.38	/
	K01959	pycA; pyruvate carboxylase subunit A	[EC:6.4.1.1]	-0.45	/
	K00170	porB; pyruvate ferredoxin oxidoreductase beta subunit	[EC:1.2.7.1]	/	-0.38
	K00239	sdhA, frdA; succinate dehydrogenase / fumarate reductase, flavoprotein subunit	[EC:1.3.5.1 1.3.5.4]	/	0.43
	K01676	E4.2.1.2A, fumA, fumB; fumarate hydratase, class I	[EC:4.2.1.2]	/	-0.39
	K01681	ACO, acnA; aconitate hydratase	[EC:4.2.1.3]	/	-0.37
	K03737	por, nifJ; pyruvate-ferredoxin/flavodoxin oxidoreductase	[EC:1.2.7.1 1.2.7.-]	/	0.43
	K18559	frdD; NADH-dependent fumarate reductase subunit D	[EC:1.3.1.6]	/	-0.45

/: no significant correlation.

Reference

Jia, Y., Wang, Q., Zhu, J., Chen, Z., He, N., and Yu, G. (2019). A spatial and temporal dataset of atmospheric inorganic nitrogen wet deposition in China (1996 – 2015). *China Scientific data* 4(1)**,** (2018-2009-2012). doi: 10.11922/sciencedb.921.

Yuan, D., and Cao, J. (2008). "Theory and practice of karst dynamics". Beijing: Chinese Science Press.

Yun, Y., Wang, H., Man, B., Xiang, X., Zhou, J., Qiu, X., et al. (2016). The relationship between pH and bacterial communities in a single karst ecosystem and its implication for soil acidification. *Front Microbiol* 7**,** 1955. doi: 10.3389/fmicb.2016.01955.
